# Supplementary material for: Trajectories of pain and cognitive function: 22 years of evidence in mid-to-later life
Source: medRxiv. 2026 Feb 13:2026.02.10.26345971. Preprint. [Version 3] doi: 10.64898/2026.02.10.26345971 (PMC12919116; doi:10.64898/2026.02.10.26345971)
Supplement: Supplement 1 [file media-1.pdf]

Supplementary material for Trajectories of pain and cognitive function: 22 years of evidence in mid-to-later life

Authors: Salomé Andres, Simon R Cox, Chloe Fawns-Ritchie

I. Distribution of the first-order variables

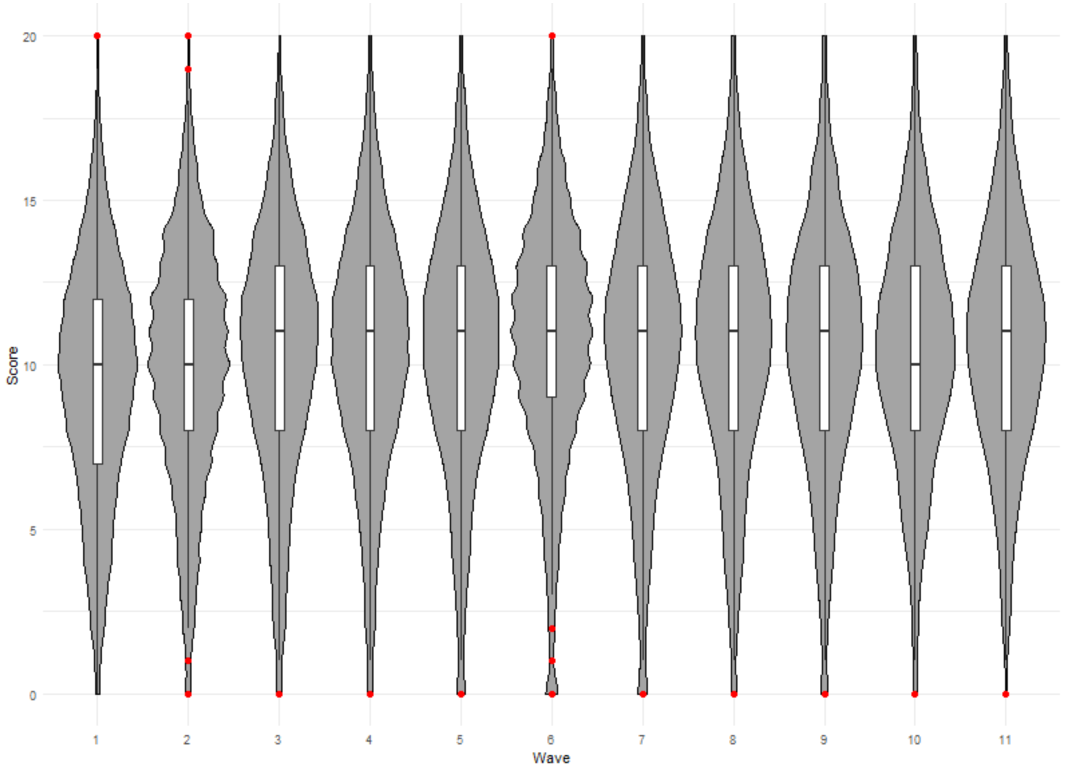

Figure S1: Distribution of scores on the word recall test (immediate and delayed summed)

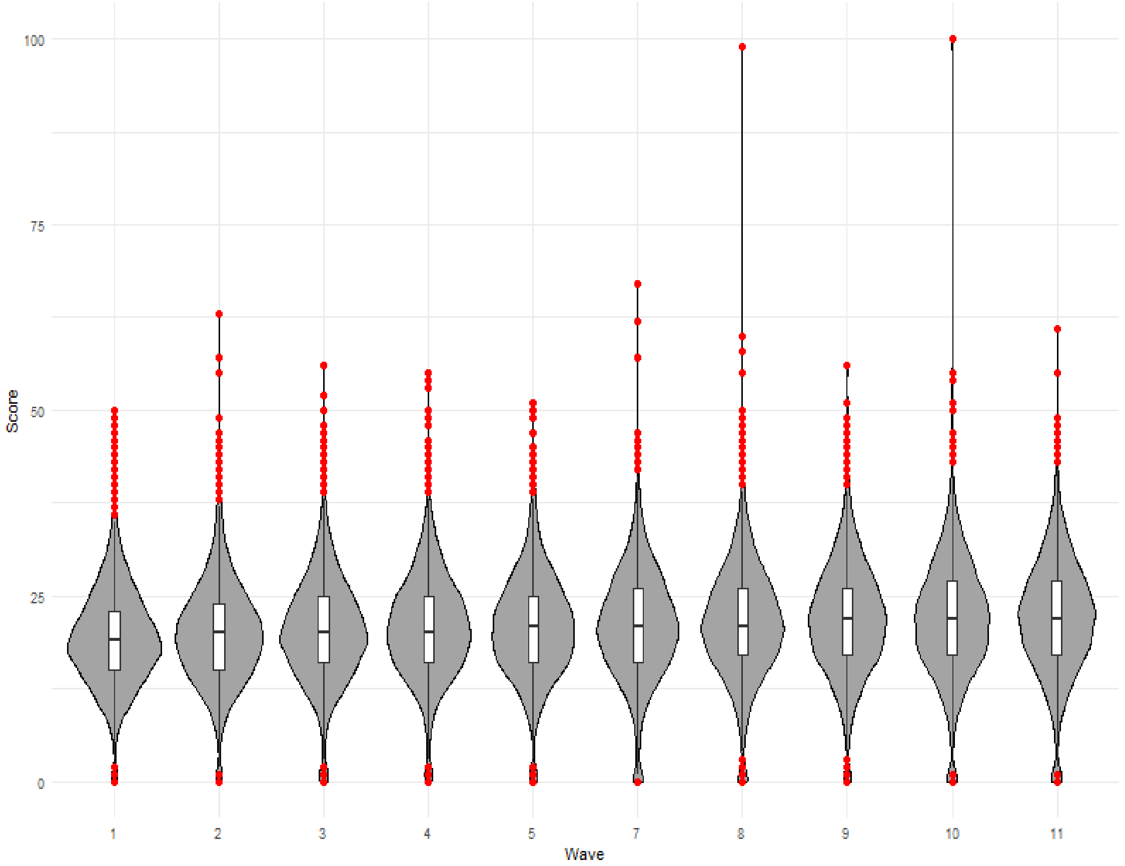

Figure S2: Distribution of scores on the animal naming test (before outliers were removed). During data cleaning, we set to NA all the results beyond 4 standard deviations (4SD = 54) as this plot demonstrated they were clear outliers. Scores of 0 were also set to NA since they likely indicate the participant did not complete or understand the task

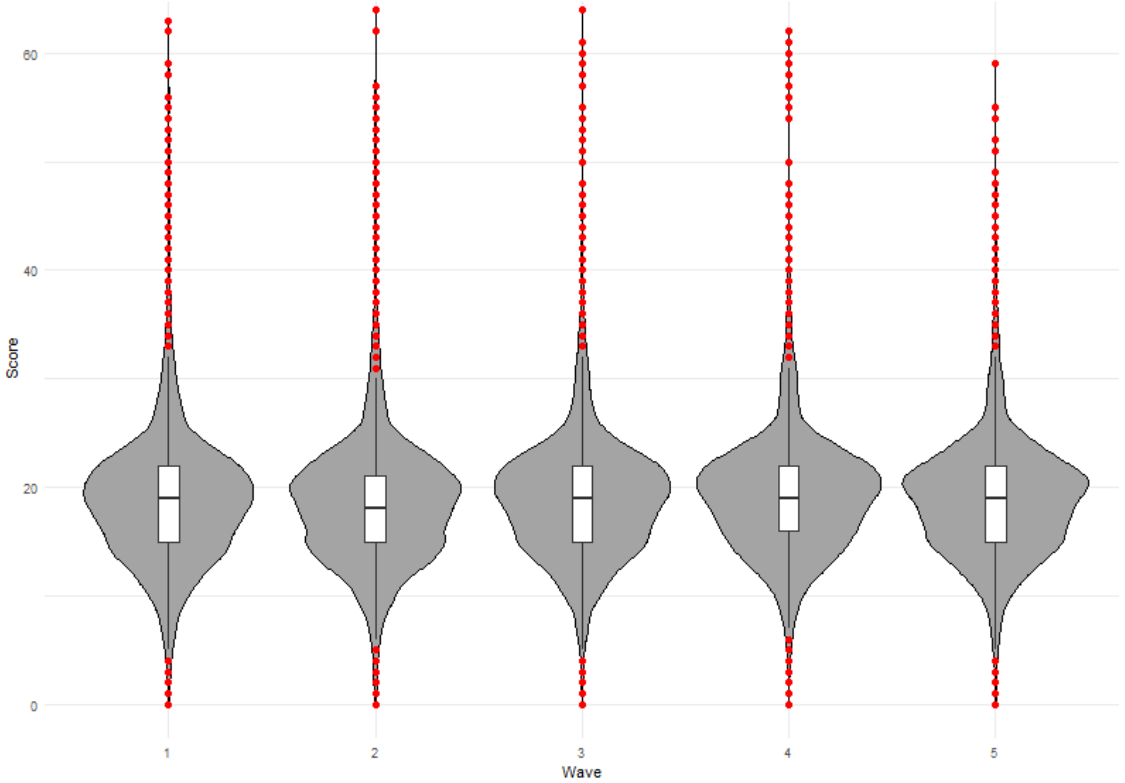

Figure S3: Distribution of scores on the letter cancellation test. During data cleaning, we set to NA all the scores reported as 0 since they likely indicate the participant did not complete or understand the task

II. Approach to building the factor-of-curves of cognitive function

To use the factor-of-curves approach, individual cognitive tests should first be more highly correlated when measured at the same wave than across waves which was demonstrable in our dataset (see correlation matrix below in Figure S4).

The next step, according to Wickrama et al.,[59] is to build a parallel process model combining all three tests where we allowed the residual variance of the latent trajectory for each cognitive test to correlate with the residual variances of the next and previous wave as well as within each domain across waves. In our model, this significantly improved fit (CFI = 0.966 → 0.983, RMSEA = 0.023 → 0.018, $p < 0.001$ on the Satorra-Bentler Scaled Chi-Square)[49] and we found stronger correlation among the slopes of the various domains rather than between the intercept and slope of each individual domain. This indicates that there is likely a global, second order growth factor we could identify, and allows us to build a factor-of-curves model using these data.

The final higher-order model built from this parallel process and used in the present study allowed the within-wave covariance of each cognitive test (at each wave, all three tests were correlated with each other); however, the test-specific covariation of intercept and slope was not supported by the data and led to a Heywood case (i.e., an impossible parameter estimate) so to allow for model convergence within boundaries, the covariance between intercept and slope of each cognitive tests was set to zero.

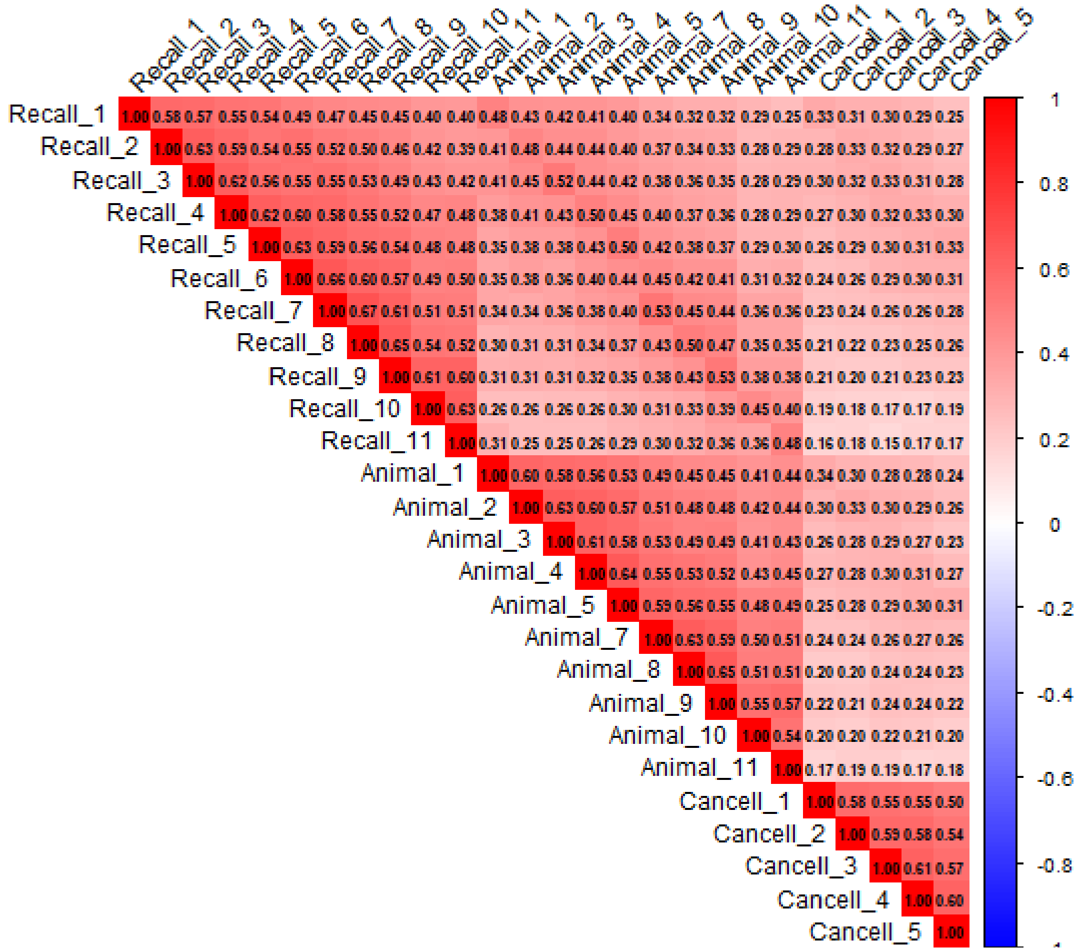

Figure S4: Correlation matrix of the cognitive tests at all time points. Animal_# = Animal naming test, Cancell_# = Letter cancellation test. This shows a higher correlation between each test among the same wave of data collection than across waves.

III. Patterns of missingness

On average there was 63% missingness on all 4 dependent variables across all waves, however, when examining the dataset wave by wave – ignoring missing data by design - this fell to 6% on average (see figures below).

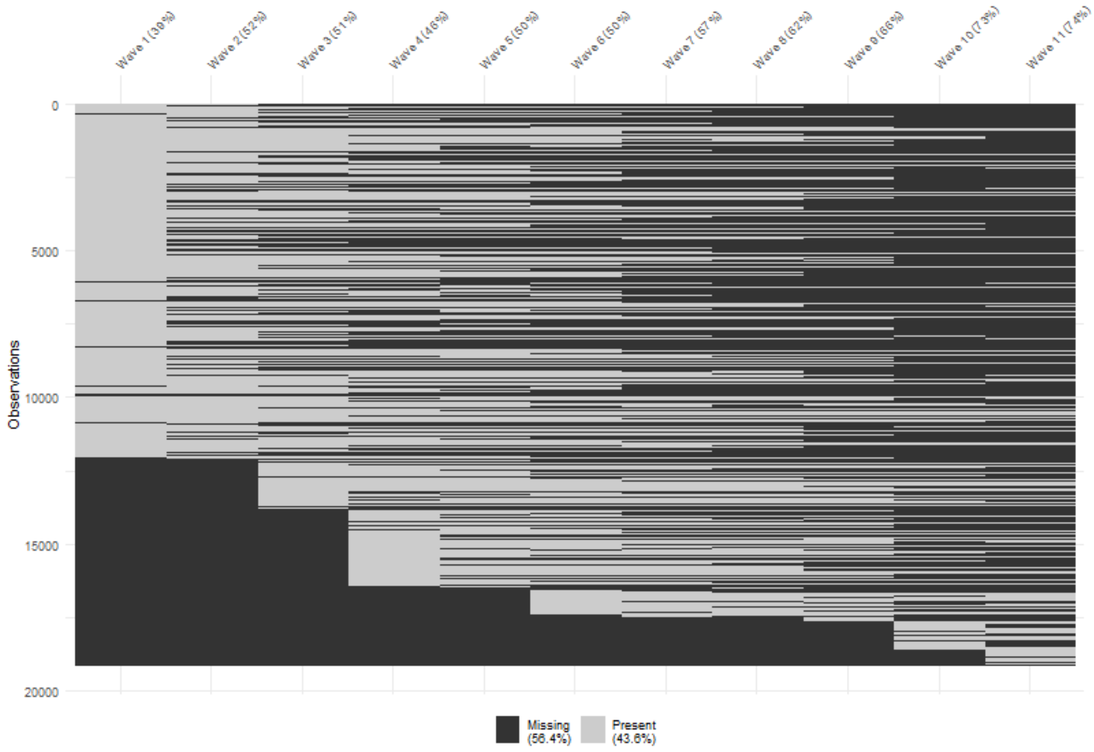

Figure S5: Patterns of missingness in the recall variable

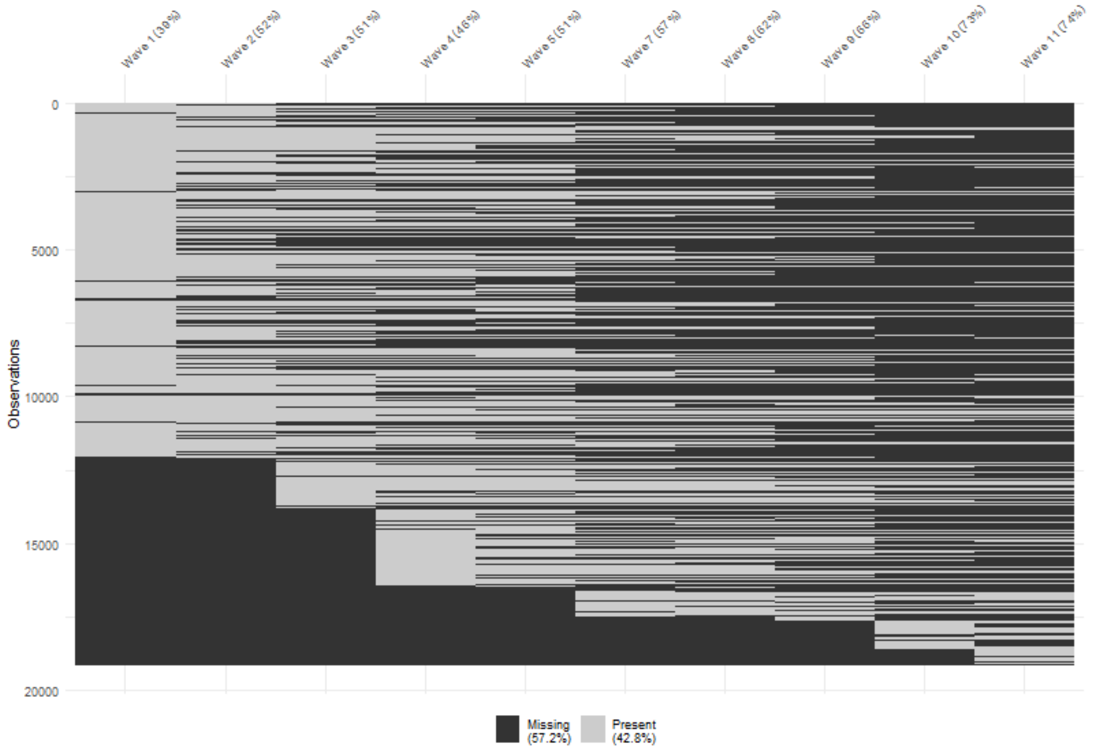

Figure S6: Patterns of missingness in the animal naming variable

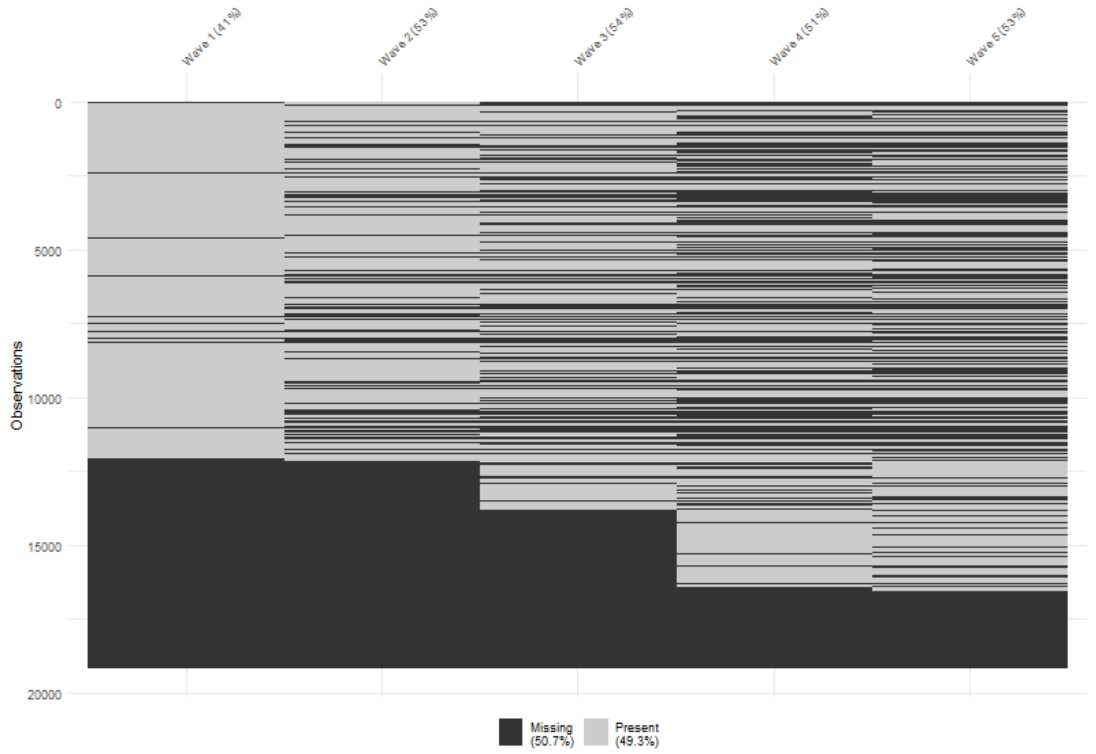

Figure S7: Patterns of missingness in the letter cancellation variable

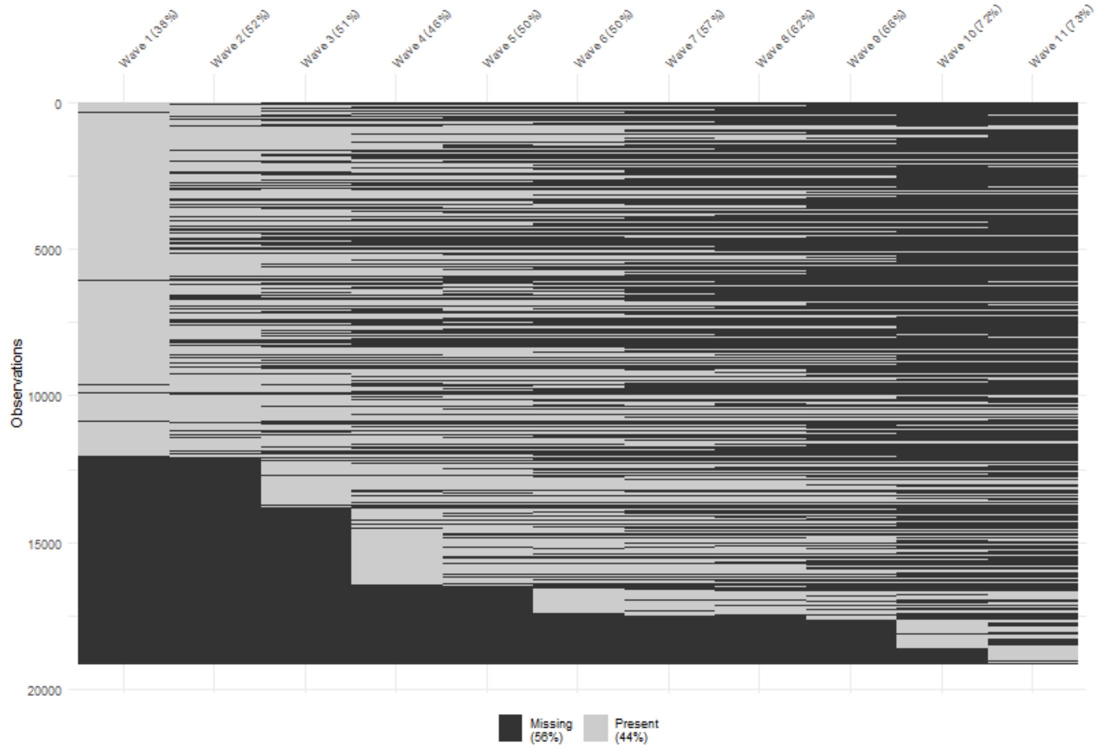

Figure S8: Patterns of missingness in the pain variable

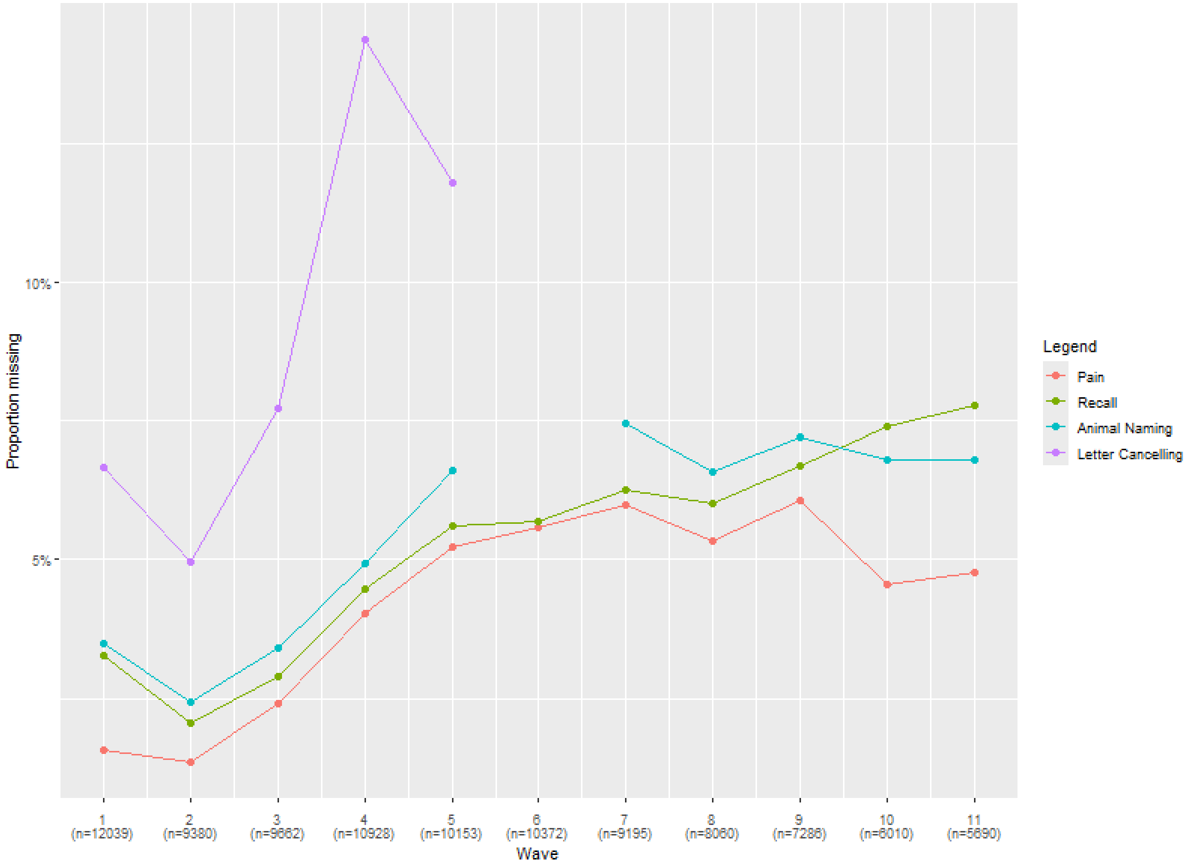

Figure S9. Proportion of missing data wave by wave

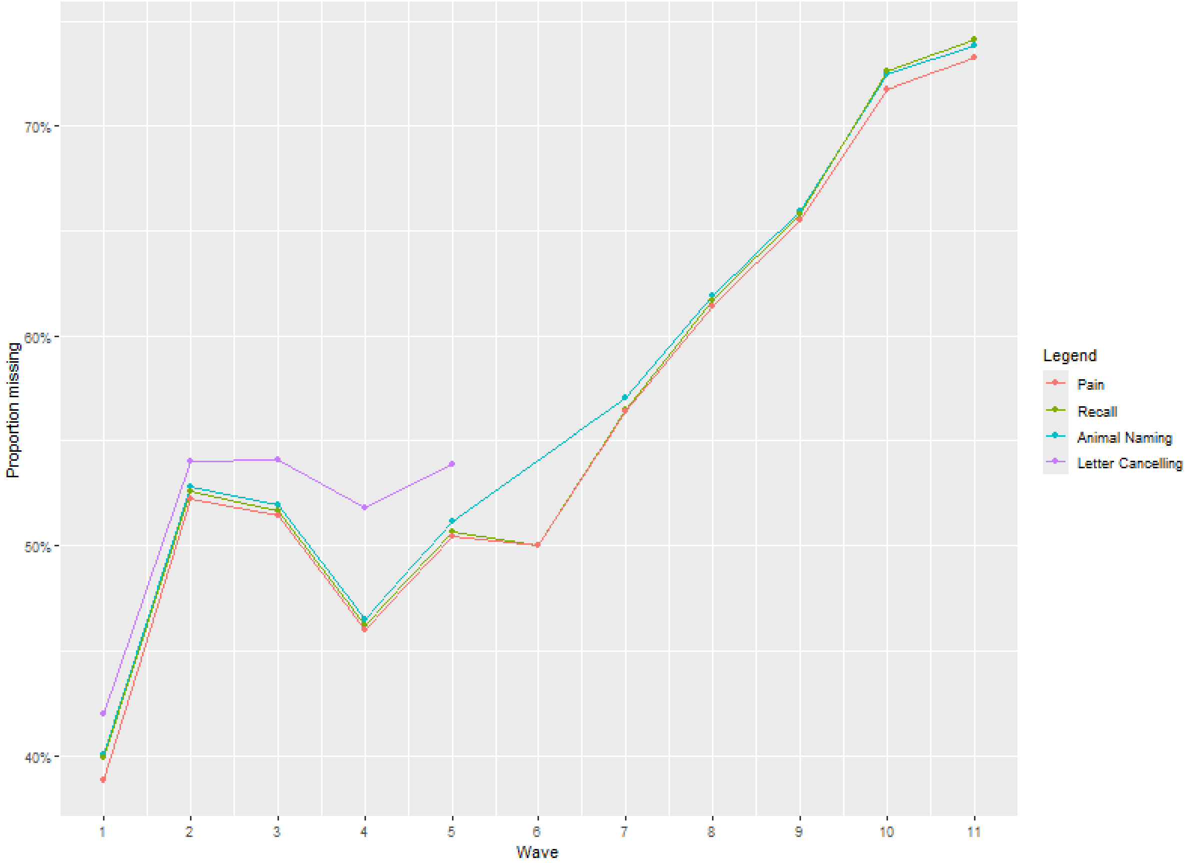

Figure S10: Proportion of missing data on full dataset (N = 19,376)

	W2	W3	W4	W5	W6	W7	W8	W9	W10	W11	Mean
0	59.82	58.36	58.85	59.50	54.38	55.44	57.61	53.23	53.55	52.99	56.37
1	9.90	10.06	10.93	10.59	12.20	12.81	11.22	12.31	11.04	11.49	11.25
2	18.32	22.20	19.83	19.77	22.11	21.88	20.26	21.48	25.15	25.40	21.64
3	11.96	9.38	10.39	10.14	11.30	9.88	10.92	12.99	10.25	10.13	10.73

Table S1: Rate of attrition at each wave of data collection for the pain variable in %. Categories 0, 1, 2, 3 represent the response to the pain variable (none, mild, moderate, severe) at the previous wave.

IV. Higher order factor loadings

Variable		Factor-of-curves LGCM			
		Estimate	SE	z-score	p value
**Loading on intercept of G**	Recall	0.896	0.007	121.895	<0.001
	Animal	0.828	0.009	94.507	<0.001
	Cancell	0.665	0.008	79.931	<0.001
**Loading on slope of G**	Recall	0.835	0.067	12.404	<0.001
	Animal	0.982	0.075	13.163	<0.001
	Cancell	0.633	0.093	6.794	<0.001
		Model 1 (No covariates)			
		Estimate	SE	z-score	p-value
**Loading on intercept of G**	Recall	0.895	0.007	130.265	<0.001
	Animal	0.831	0.008	103.367	<0.001
	Cancell	0.664	0.008	80.06	<0.001
**Loading on slope of G**	Recall	0.851	0.053	15.959	<0.001
	Animal	0.962	0.057	16.839	<0.001
	Cancell	0.632	0.091	6.946	<0.001
		Model 2 (Age and sex)			
		Estimate	SE	z-score	p value
**Loading on intercept of G**	Recall	0.925	0.006	161.867	<0.001
	Animal	0.787	0.007	109.179	<0.001
	Cancell	0.669	0.009	74.445	<0.001
**Loading on slope of G**	Recall	0.909	0.019	46.971	<0.001
	Animal	0.899	0.021	42.433	<0.001
	Cancell	0.725	0.091	7.943	<0.001
		Model 3 (Age, sex, ethnicity, socio-economic status and comorbidities)			
		Estimate	SE	z-score	p-value
**Loading on intercept of G**	Recall	0.916	0.005	173.553	<0.001
	Animal	0.802	0.007	107.407	<0.001
	Cancell	0.669	0.009	78.477	<0.001
**Loading on slope of G**	Recall	0.914	0.023	39.614	<0.001
	Animal	0.885	0.026	34.591	<0.001
	Cancell	0.73	0.091	8.026	<0.001

Table S2: Loadings on higher order factor from each cognitive test in the LGCM (before adding pain to the model) and 3 parallel process models. G = General cognitive function higher order factor, Recall = Word Recall test, Animal = Animal naming test, Cancell = Letter cancellation test, SE = Standard error. P-values have not undergone FDR correction.

V. Path diagram of the parallel process of pain on individual cognitive test

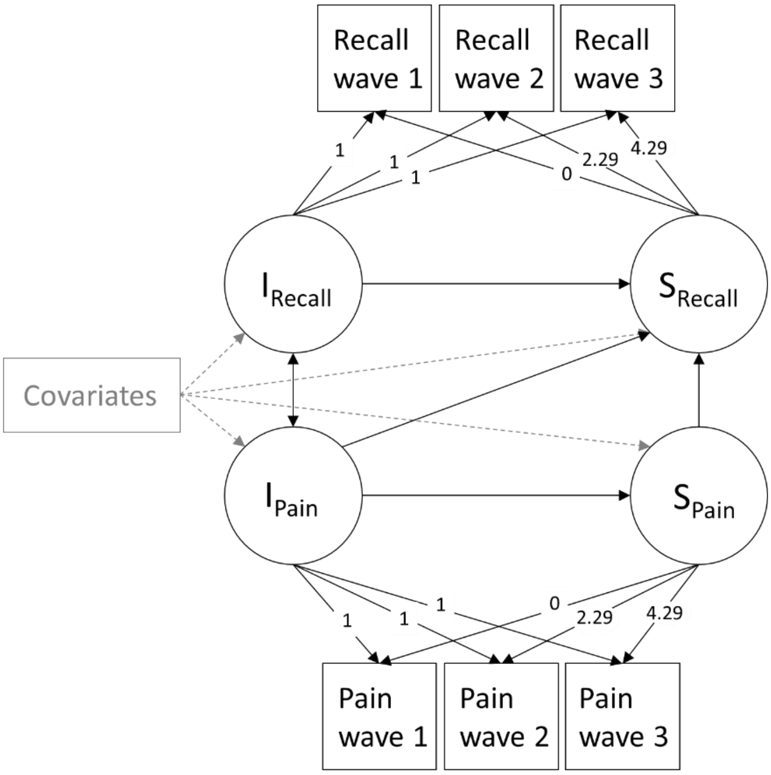

Figure 1S11: Simplified path diagram of the parallel process latent growth curve model of pain severity and a single cognitive test (word recall for illustrative purposes). Circles are latent factors, squares represent observed variables, single-headed arrows are regression paths and double-headed arrows are correlation paths. Dashed grey lines are regression paths for covariates. I = Intercept, S = Slope, Recall = Word recall test. For illustrative purposes, only the first three waves are shown, but all 11 waves were used in the model.

VI. Descriptive statistics on covariates

Wave number	1	2	3	4	5	6	7	8	9	10	11
Years since wave 1	0	2.29	4.29	6.25	8.29	10.21	12.21	14.21	16.29	19.79	21.59
N	12039	9380	9662	10928	10153	10372	9195	8060	7286	6010	5690
**Sex (Female)**											
N	6716	5267	5398	6033	5616	5693	5065	4448	4013	3230	3083
%	55.8	56.2	55.9	55.2	55.3	54.9	55.1	55.2	55.1	53.7	54.2
**Age**											
N	12037	9380	9662	10928	10153	10372	9195	8060	7286	6010	5550
Mean	64.27	65.86	64.74	65.41	66.97	66.97	68.28	69.75	71.02	71.50	72.15
SD	10.78	10.31	10.92	10.01	9.63	9.83	9.47	8.92	8.62	8.04	7.21
**Ethnicity (White)**											
N	11601	9145	9365	10553	9808	9979	8846	7774	7015	5713	5342
%	98.4	97.6	97.0	96.7	96.6	96.2	96.2	96.5	96.3	95.4	94.2
**Socioeconomic Status**											
SES 1											
N	1042	834	907	1041	1019	1096	964	845	789	773	869
%	8.8	9.0	9.5	9.9	10.3	10.8	10.9	10.8	11.3	13.5	15.6
SES 2											
N	2077	2019	2164	2490	2365	2436	2128	1897	1710	1519	1522
%	17.6	21.7	22.6	23.7	23.9	24.1	24.1	24.3	24.5	26.5	27.4
SES 3											
N	2067	1299	1330	1430	1366	1377	1252	1113	992	812	819
%	17.5	13.9	13.9	13.6	13.8	13.6	14.2	14.3	14.2	14.2	14.7
SES 4											
N	1184	978	1069	1223	1157	1187	1099	967	885	723	568
%	10.1	10.5	11.2	11.6	11.7	11.7	12.4	12.4	12.7	12.6	10.2
SES 5											
N	1361	994	1003	1019	954	928	776	659	584	393	336
%	11.6	10.7	10.5	9.7	9.6	9.2	8.8	8.4	8.4	6.9	6.0
SES 6											
N	2153	1667	1642	1805	1652	1695	1458	1303	1132	838	697
%	18.3	17.9	17.2	17.2	16.7	16.8	16.5	16.7	16.2	14.6	12.5
SES 7											
N	1897	1381	1314	1371	1288	1300	1086	956	826	616	602
%	16.1	14.8	13.8	13.1	13.0	12.9	12.3	12.3	11.9	10.8	10.8
SES 8											
N	0	144	126	120	99	94	76	60	50	48	150
%	0.0	1.5	1.3	1.1	1.0	0.9	0.9	0.8	0.7	0.8	2.7
**Comorbidities***											
Diabetes											
N	864	383	255	377	288	308	259	193	263	819	246
%	7.2	4.1	2.6	3.5	2.8	3.0	2.8	2.4	3.6	13.6	4.3
Hypertension											
N	4465	1704	914	1282	772	802	593	469	489	2874	755
%	37.1	18.2	9.5	11.7	7.6	7.7	6.5	5.8	6.7	47.9	13.3
Heart disease											
N	2156	851	491	726	617	611	667	576	632	1522	507
%	17.9	9.1	5.1	6.7	6.1	5.9	7.3	7.2	8.7	25.3	8.9
Lung disease											
N	762	687	129	244	140	179	145	114	107	434	138
%	6.3	7.3	1.3	2.2	1.4	1.7	1.6	1.4	1.5	7.2	2.4
Stroke											
N	511	166	107	176	148	154	134	148	105	363	94
%	4.2	1.8	1.1	1.6	1.5	1.5	1.5	1.8	1.4	6.0	1.7
Cancer											

N	727	703	233	346	304	303	279	268	242	981	233
%	6.0	7.5	2.4	3.2	3.0	2.9	3.0	3.3	3.3	16.3	4.1

Table S3: Descriptive statistics for covariates across all 11 waves. SES = Socioeconomic Status based on the National Statistics Socio-Economic Classification.[25] *At wave 10, the question "Diagnosed [condition] newly reported" changed to "Whether ever been told had [condition] by doctor". This had no impact on our analysis since we adjusted the third model for comorbidities in a time-invariant fashion and considered the participants as having a condition if they ever reported it, at any wave of the dataset.

VII. Individual and mean trajectories for each cognitive test

Figure S12: Trajectories of recall test results. The unstandardised estimates of the word recall test intercept and slope from a random sample of 150 participants estimated by measurement model LGCM are shown as well as the mean unstandardised estimate from the model in dark red (intercept β = 10.320 and slope β = -0.064)

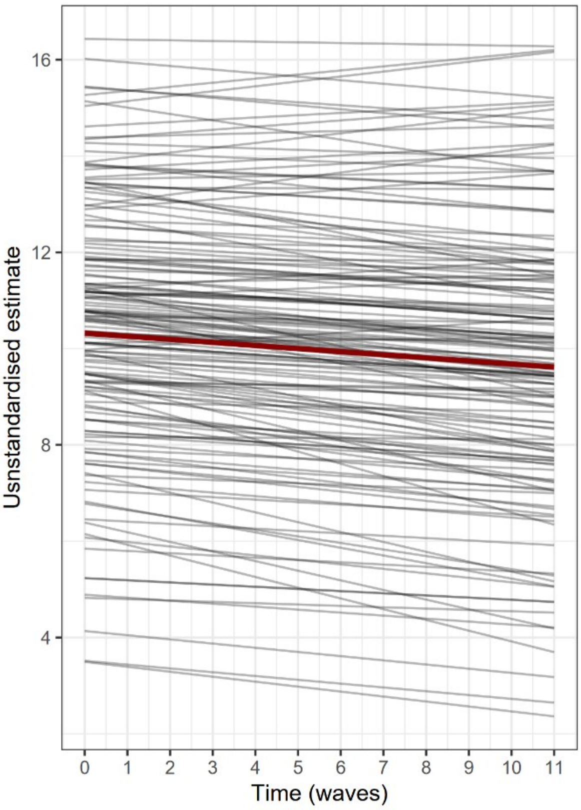

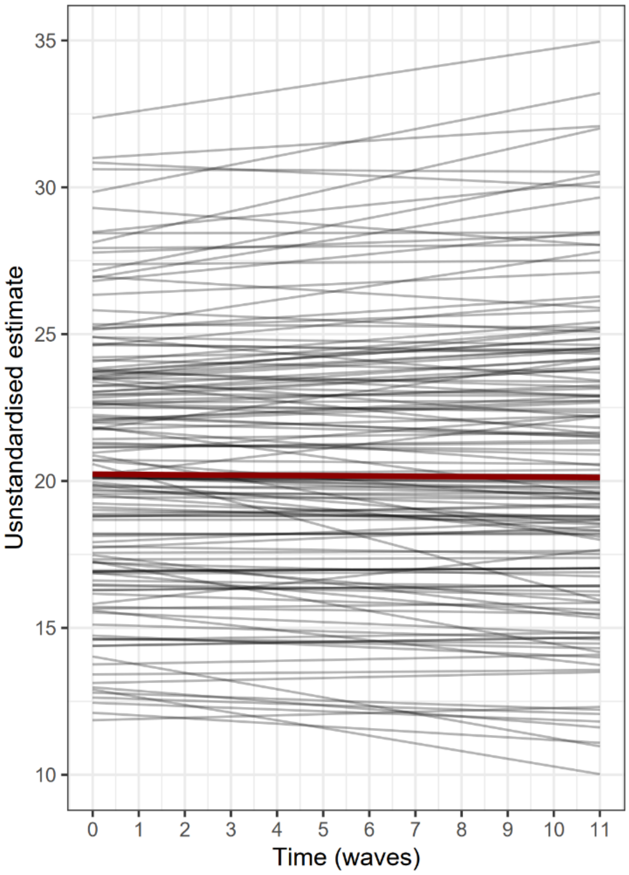

Figure S13 Trajectories of animal naming test results. The unstandardised estimates of the animal naming test intercept and slope from a random sample of 150 participants estimated by measurement model LGCM are shown as well as the mean unstandardised estimate from the model in dark red (intercept β = 20.231 and slope β = -0.010)

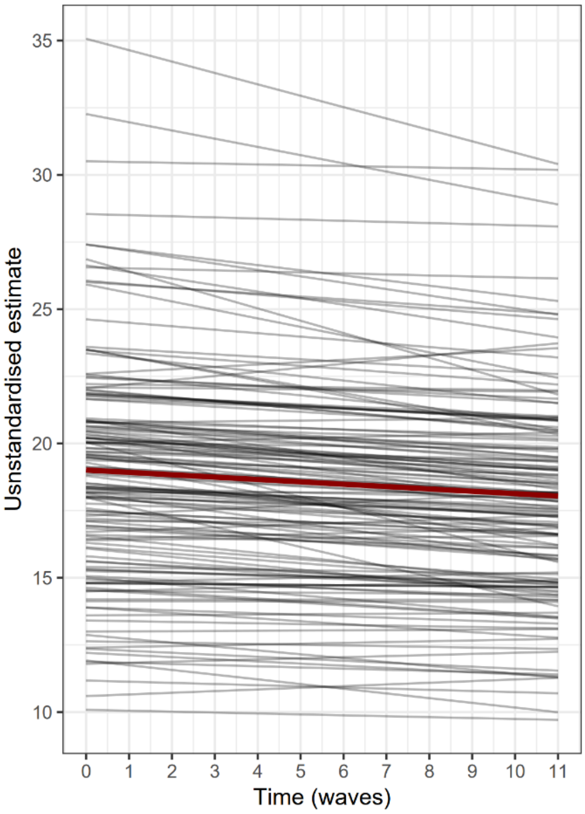

Figure S14: Trajectories of letter cancellation test results. The unstandardised estimates of letter cancellation test results intercept and slope from a random sample of 150 participants estimated by measurement model LGCM are shown as well as the mean unstandardised estimate from the model in dark red (intercept β = 19.012 and slope β = -0.088)

VIII. Rank order stability analysis

Rank-order stability was investigated for the four observed dependent variables (pain and three cognitive tests) and found to be high on all outcomes. To allow equal-length comparisons across measures we focussed on rank-order stability over waves 1–5, 5–10 and 1-10 and letter cancellation is reported on 1-3, 3-5 and 1-5 due to the lack of measurement beyond wave 5. The model-implied correlations between latent true scores during the first half of the study (at waves 1 to 5) were large: pain r = 0.934, recall r = 0.945 and animal naming r = 0.938. Stability over the second half (at waves 5 to 10) remained high: pain r = 0.873, recall r = 0.929, animal naming r = 0.918. Finally, overall rank order from waves 1 to 10 was still very stable: pain r = 0.641, recall r = 0.755 and animal naming r = 0.723. A similar pattern was found for the letter cancellation test: waves 1 to 3 r = 0.966, waves 3 to 5 r = 0.969 and waves 1 to 5 r = 0.874. These results are in concordance with the high variance in intercept and low variance in slopes for all four observed dependent variables, indicating that while participants differed in their initial levels of reported pain and measured cognitive function, their rates of change are similar, often maintaining the order in which they began the study.

IX. Regression coefficients on covariates

Variable		Model 2 (Age and sex)				Model 3 (Age, sex, ethnicity, socio-economic status and comorbidities)			
		Estimate	SE	z-score	p value	Estimate	SE	z-score	p-value
**Intercept of G regressed on**	Age	-0.598	0.008	-75.956	<0.001	-0.562	0.008	-70.040	<0.001
	Sex (Female)	0.086	0.010	8.930	<0.001	0.113	0.010	11.632	<0.001
	Ethnicity (White)					0.168	0.011	14.986	<0.001
	SES					-0.339	0.008	-44.457	<0.001
	Diabetes					-0.013	0.008	-1.715	0.086
	Hypertension					-0.046	0.008	-5.713	<0.001
	Cancer					0.029	0.007	3.830	<0.001
	Heart disease					0.006	0.008	0.766	0.443
	Lung disease					-0.022	0.008	-2.903	0.004
	Stroke					-0.044	0.008	-5.212	<0.001
**Slope of G regressed on**	Age	-0.617	0.019	-33.021	<0.001	-0.646	0.019	-34.469	<0.001
	Sex (Female)	0.025	0.020	1.253	0.210	0.034	0.022	1.549	0.121
	Ethnicity (White)					0.050	0.017	2.856	0.004
	SES					-0.046	0.018	-2.547	0.011
	Diabetes					-0.027	0.014	-1.967	0.049
	Hypertension					0.001	0.015	0.087	0.931
	Cancer					-0.002	0.014	-0.179	0.858
	Heart disease					0.005	0.014	0.349	0.727
	Lung disease					0.001	0.015	0.100	0.920
	Stroke					-0.059	0.016	-3.662	<0.001
**Intercept of pain regressed on**	Age	0.116	0.011	10.888	<0.001	0.080	0.011	7.336	<0.001
	Sex (Female)	0.088	0.010	8.737	<0.001	0.080	0.010	7.966	<0.001
	Ethnicity (White)					-0.032	0.012	-2.760	0.006
	SES					0.201	0.010	20.261	<0.001
	Diabetes					0.063	0.010	6.362	<0.001
	Hypertension					-0.006	0.011	-0.550	0.582
	Cancer					0.005	0.010	0.533	0.594
	Heart disease					0.085	0.010	8.486	<0.001
	Lung disease					0.078	0.010	7.914	<0.001
	Stroke					0.007	0.010	0.679	0.497
**Slope of pain regressed on**	Age	0.092	0.020	4.586	<0.001	0.076	0.020	3.780	<0.001
	Sex (Female)	0.106	0.015	6.889	<0.001	0.108	0.015	7.008	<0.001
	Ethnicity (White)					-0.007	0.016	-0.422	0.673
	SES					0.072	0.016	4.511	<0.001
	Diabetes					0.010	0.016	0.617	0.537
	Hypertension					0.027	0.017	1.597	0.110
	Cancer					0.020	0.015	1.340	0.180
	Heart disease					0.052	0.015	3.505	<0.001
	Lung disease					0.036	0.016	2.217	0.027
	Stroke					0.055	0.017	3.274	0.001

Table S4: Coefficient of covariates in model 2 and 3. G = General cognitive function higher-order factor, SES = Socioeconomic status and SE = Standard error. P-values have not undergone FDR correction

X. Model fit information for the 4 parallel process models

Model	Covariates	AIC	BIC	Sample-size adjusted BIC
Parallel process LGCM of pain and general cognitive function	None	1410120.278	1410795.022	1410521.718
	Sex and Age	1400981.591	1401719.007	1401420.280
	All	1386863.319	1387849.076	1387448.655
Parallel process LGCM of pain and word recall	None	632387.659	632677.956	632560.372
	Sex and Age	624450.573	624803.591	624660.583
	All	616124.612	616727.019	616482.318
Parallel process LGCM of pain and animal naming	None	684830.888	685113.334	684998.927
	Sex and Age	679854.933	680200.099	680060.269
	All	671526.027	672120.611	671879.087
Parallel process LGCM of pain and letter cancellation	None	455865.962	456109.177	456010.660
	Sex and Age	451791.351	452097.292	451973.352
	All	446955.809	447511.275	447285.641

Table S5: Akaike information criteria (AIC), Bayesian information criteria (BIC) and sample-size adjusted BIC for the 4 main models of analysis derived across 3 levels of covariate adjustment

XI. Longitudinal association between pain and individual cognitive tests

Nine models were run: three for the word-recall test, three for the animal naming test and three for the letter cancellation test. Each set of three models followed the structure of the primary analysis with no covariates included in model 1, adjustment for age and sex in model 2 and full adjustment for all covariates in model 3. As before, the primary associations of interest were the regression path from latent slope and latent intercept of pain to latent slope of the cognitive test. Table S6 reports the results for the relationship between pain severity and individual cognitive test intercepts and slopes. Overall, the direction, size, and significance of effects are similar for the word-recall and animal naming tests, compared to the factor-of-curves models, but effects differ on the letter cancellation test.

For letter cancellation, there was a small negative correlation between the intercept of pain and the intercept of letter cancellation in the unadjusted model (M1, Table 3). This correlation was slightly attenuated but remained significant in the fully adjusted model (M3, Table 3). In contrast to general cognitive function, the recall test and the animal naming test, the associations between intercept and slope of pain with slope of letter cancellation were small and non-significant even in the unadjusted model, and remained so with adjustment for covariates (Table 3).

Inspection of the 95% confidence intervals confirmed the equivalence between the results of the word recall and animal naming models and the disparity with the letter cancellation models.

	Model 1 (no covariates)			Model 2 (age and sex)			Model 3 (age, sex, ethnicity, socio-economic status and comorbidities)		
Variables	β	SE	*p*	β	SE	*p*	β	SE	*p*
**Word recall**									
Slope of pain → Slope of recall	-0.099	0.029	0.001	-0.065	0.027	0.018	-0.033	0.027	0.328
Intercept of pain → Slope of recall	-0.113	0.020	<0.001	-0.112	0.020	<0.001	-0.093	0.020	<0.001
Intercept of recall → Slope of recall	-0.113	0.024	<0.001	-0.192	0.027	<0.001	-0.232	0.029	<0.001
Intercept of pain → Slope of pain	-0.414	0.023	<0.001	-0.433	0.023	<0.001	-0.462	0.025	<0.001
Intercept of recall ↔ Intercept of pain	-0.231	0.011	<0.001	-0.211	0.012	<0.001	-0.134	0.013	<0.001
**Animal naming**									
Slope of pain → Slope of animal	-0.083	0.028	0.004	-0.038	0.026	0.148	-0.013	0.026	0.696
Intercept of pain → Slope of animal	-0.148	0.020	<0.001	-0.120	0.019	<0.001	-0.104	0.019	<0.001
Intercept of animal → Slope of animal	-0.168	0.021	<0.001	-0.208	0.022	<0.001	-0.228	0.024	<0.001
Intercept of pain → Slope of pain	-0.415	0.023	<0.001	-0.432	0.023	<0.001	-0.462	0.023	<0.001
Intercept of animal ↔ Intercept of pain	-0.214	0.011	<0.001	-0.172	0.012	<0.001	-0.102	0.012	<0.001
**Letter cancellation**									
Slope of pain → Slope of cancell	0.022	0.043	0.608	-0.008	0.042	0.847	0.005	0.042	0.908
Intercept of pain → Slope of cancell	-0.045	0.029	0.133	-0.048	0.029	0.111	-0.043	0.030	0.171
Intercept of cancell → Slope of cancell	-0.305	0.034	<0.001	-0.389	0.038	<0.001	-0.386	0.040	<0.001
Intercept of pain → Slope of pain	-0.418	0.023	<0.001	-0.433	0.023	<0.001	-0.462	0.023	<0.001
Intercept of cancell ↔ Intercept of pain	-0.136	0.013	<0.001	-0.123	0.014	<0.001	-0.075	0.014	<0.001

Table S6: Summary of results for individual test associations. Estimates are all standardised. β = Estimate, SE = standard error, *p* = p-value, recall = word recall test, animal = animal naming test, cancell = letter cancellation test. → represent regression paths, ↔ show correlations. The p-values are adjusted within each model for false discovery rate using the Benjamini–Hochberg procedure with α = 0.05.

XII. Bivariate models

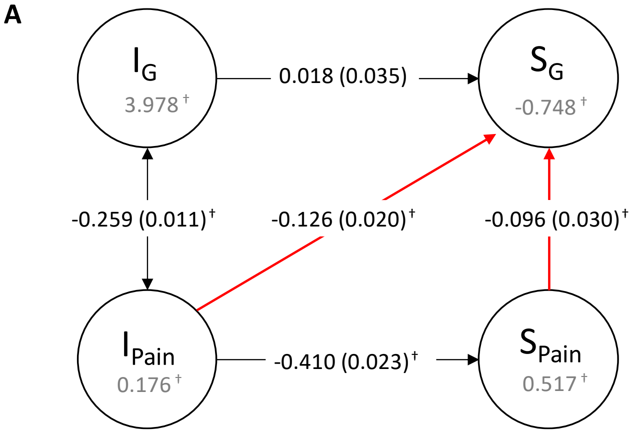

Figure S15: Side-by-side comparison of original model with bivariate analyses. A is the original model (model 1 reported in main text); B is running the regression of intercept of pain on slope of cognitive function without the mediating effect of slope of pain; C shows the association between slope of pain and slope of cognitive function without its conditional relationship with intercept of pain

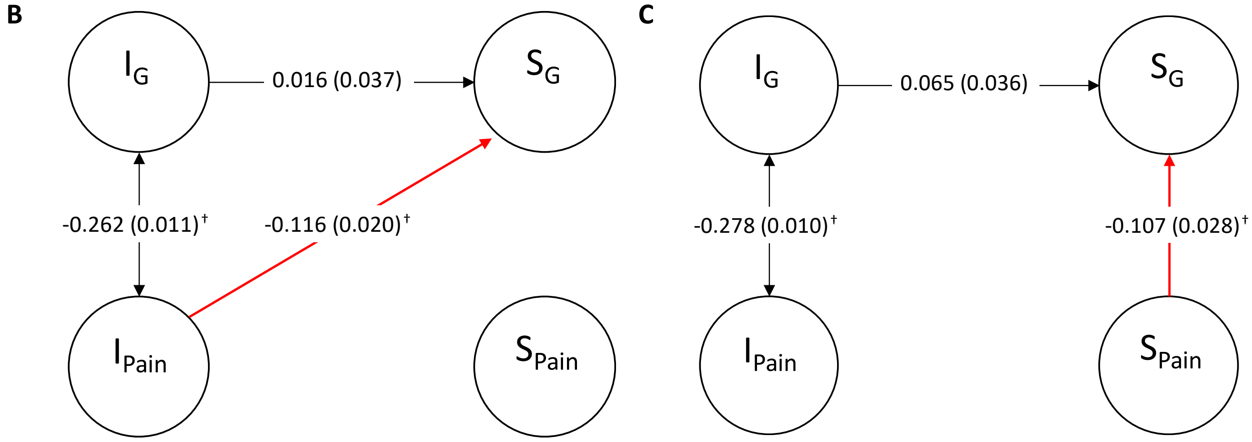

XIII. Supplementary sensitivity analyses

We ran the same models as in the primary analysis but without partial invariance of the thresholds. For these three models, MPlus indicates that the third threshold parameter of the pain variable had to be fixed to avoid singularity of the information matrix. We found similar regression and correlation coefficients to the primary analysis models but some discrepancies in the estimated means of intercept and slope of pain severity. The estimates and FDR-adjusted p-values for the three models built under these conditions are in Table S7.

	Model 1 (no covariates)			Model 2 (Age and sex)			Model 3 (Age, sex, ethnicity, socio-economic status and comorbidities)		
Variables	Estimate	SE	*p*	Estimate	SE	*p*	Estimate	SE	*p*
**Regressions**									
Slope of g → Slope of pain	-0.102	0.031	0.002	-0.053	0.027	0.063	-0.024	0.027	0.387
Slope of g → Intercept of pain	-0.136	0.021	<0.001	-0.121	0.018	<0.001	-0.105	0.019	<0.001
Slope of g → Intercept of g	0.018	0.035	0.614	-0.145	0.031	<0.001	-0.207	0.036	<0.001
Slope of pain → Intercept of pain	-0.474	0.019	<0.001	-0.495	0.019	<0.001	-0.523	0.020	<0.001
**Correlation**									
Intercept of g ↔ Intercept of pain	-0.256	0.011	<0.001	-0.238	0.012	<0.001	-0.154	0.013	<0.001
**Means**									
Intercept of g	3.978	0.045	<0.001	3.821	0.043	<0.001	3.714	0.146	<0.001
Slope of g	-0.648	0.204	0.002	-0.459	0.133	0.001	-0.289	0.022	0.170
Intercept of pain	0.855	0.022	<0.001	-0.768	0.016	<0.001	-0.430	0.093	<0.001
Slope of pain	0.845	0.029	<0.001	0.015	0.029	0.604	0.182	0.005	0.044

Table S7: Models without partial time-invariance of the thresholds: Estimates are all standardised. SE = Standard Error. g = general cognitive function as determined by the factor-of-curves method. The p-values are adjusted within each model for false discovery rate using the Benjamini–Hochberg procedure with a = 0.05.

Another set of models was run using only waves 1 to 9 to investigate whether the results of the main models are consistent with results using only data collected before the COVID-19 pandemic. This was done because the average interval and the individual-person intervals after wave 9 are much more varied when compared to the relatively consistent intervals between waves 1 to 9. Like the models in the main analysis, these three models' loadings on the first-order factors were set to the mean time (in years) elapsed since wave 1 and the thresholds of the pain variable were partially invariant. The regression and correlation paths were very similar to the primary models. The main difference with previous results lies again with the estimated means of intercept and slope of pain severity. The results of these models are shown in Table S8.

	Model 1 (no covariates)			Model 2 (Age and sex)			Model 3 (Age, sex, ethnicity, socio-economic status and comorbidities)		
Variables	Estimate	SE	*p*	Estimate	SE	*p*	Estimate	SE	*p*
**Regressions**									
Slope of g → Slope of pain	-0.108	0.031	0.001	-0.073	0.026	0.006	-0.046	0.026	0.085
Slope of g → Intercept of pain	-0.123	0.020	<0.001	-0.112	0.018	<0.001	-0.102	0.018	<0.001
Slope of g → Intercept of g	0.090	0.030	0.003	-0.105	0.031	0.001	-0.158	0.035	<0.001
Slope of pain → Intercept of pain	-0.386	0.026	<0.001	-0.401	0.026	<0.001	-0.428	0.026	<0.001
**Correlation**									
Intercept of g ↔ Intercept of pain	-0.253	0.011	<0.001	-0.235	0.012	<0.001	-0.145	0.014	<0.001
**Means**									
Intercept of g	3.987	0.048	<0.001	3.835	0.046	<0.001	3.723	0.064	<0.001
Slope of g	-0.751	0.150	<0.001	-0.561	0.124	<0.001	0.321	0.199	0.107
Intercept of pain	0.563	0.032	<0.001	-3.151	0.052	<0.001	3.359	0.093	<0.001
Slope of pain	0.570	0.030	<0.001	-0.948	0.071	<0.001	1.870	0.132	<0.001

Table S8: Models on waves 1-9 (before the COVID-19 pandemic): Estimates are all standardised. SE = Standard Error. g = general cognitive function as determined by the factor-of-curves method. The p-values are adjusted within each model for false discovery rate using the Benjamini–Hochberg procedure with a = 0.05.
